# New data on spermiogenesis and trepaxonematan axoneme in basal tapeworms (Cestoda, Caryophyllidea, Lytocestidae) parasitizing cyprinid fishes

**DOI:** 10.1038/s41598-019-49312-9

**Published:** 2019-09-09

**Authors:** Martina Matoušková, Tomáš Bílý, Magdaléna Bruňanská, Mikuláš Oros, Borislav Kostič, Jana Nebesářová

**Affiliations:** 1 Institute of Parasitology, Slovak Academy of Sciences, Košice, Slovak Republic; 2 Institute of Parasitology, Biology Centre of the Czech Academy of Sciences, České Budějovice, Czech Republic; 3 Faculty of Science, University of South Bohemia, České Budějovice, Czech Republic

**Keywords:** Differentiation, Ichthyology

## Abstract

Monozoic caryophyllidean cestodes, intestinal parasites of cyprinid fishes, represent a group of tapeworms with an unclear evolutionary history. As spermatology may provide phylogenetically important data, the spermiogenesis and ultrastructure of the mature spermatozoon have been investigated using an integrative approach combining transmission electron microscopy, cytochemistry and electron tomography in *Khawia rossittensis* (Szidat, 1937). The process of spermatid formation is accompanied by the presence of ultrastructural characters not described in traditional models of spermiogenesis, e.g., apical electron-dense material, the two striated roots situated unusually opposite each other, branching of typical striated roots, an intercentriolar body comprising five electron-dense and four electron-lucent layers, rotation of both free flagella and flagellar buds to the median cytoplasmic process at 90°, and a complete proximodistal fusion. The synchronous rotation of both flagellar buds and growing free flagella is an evolutionarily linked pattern favouring the hypothesis that the Caryophyllidea are not ancestral but are secondarily derived from polyzoic forms. Electron tomography analysis has revealed a unique feature of two helicoidal tubular structures in the central electron-dense core of the axoneme of mature spermatozoon. These data provide new insights into the architecture of the 9 + ‘1’ axoneme, which is shared by male gametes of all trepaxonematan Platyhelminthes.

## Introduction

Caryophyllidean tapeworms are monozoic, i.e., without external and internal proglottidation, and contain only a single set of reproductive organs; there are 42 genera and 122 valid species distributed in four families^1^. This group apparently played a key role in the evolution of cestodes^2^ and represents a basal or nearly basal group of tapeworms; however, phylogenetic relationships within the order Caryophyllidea and its evolutionary interrelationships with the most relative group of tapeworms remain unsolved^3,4^.

Sperm characters provide especially important datasets useful for phylogeny^5–7^. Although the database of existing spermatological characters of cestodes has been considerably extended in the last decade^8^, substantial gaps in the knowledge on the spermatology of the most basal tapeworm groups persist^9^. Of the 12 species of the order Caryophyllidea that have been the subject of ultrastructural spermatological studies, 6 species belong to the family Lytocestidae, namely, *Atractolytocestus huronensis*^10^, *Caryophyllaeides fennica*^11^, *Khawia armeniaca*^12^, *Khawia sinensis*^13^, *Lytocestus indicus*^14^, *Monobothrioides chalmersius*^15^.

The basic sperm structure of the trepaxonematan type 9 + ‘1’ (see Ehlers^16^) has been analysed using electron tomography in a recent pilot study of the lytocestid caryophyllidean tapeworm *Caryophyllaeides fennica*^11^. The results showed for the first time that two tubular structures are present in the central axonemal electron-dense core in Trepaxonemata. The helicoidal nature of the centre of the complex axonemal core has been reported using electron tomography analysis in one-axoneme spermatozoa of the evolutionarily more derived tapeworm *Nippotaenia mogurndae*^17^. These interesting findings call for further detailed electron tomography studies of other caryophyllidean cestodes and cestode groups to elucidate the basic sperm structure of type 9 + ‘1’ in the Trepaxonemata.

Therefore, the spermatological characteristics of *Khawia rossittensis*, another lytocestid tapeworm and parasite of *Carassius* *gibelio*, have been examined to shed more light on the cytodifferentiation and fine structural architecture of the male gametes of caryophyllidean cestodes.

## Results

**Figure 1:**
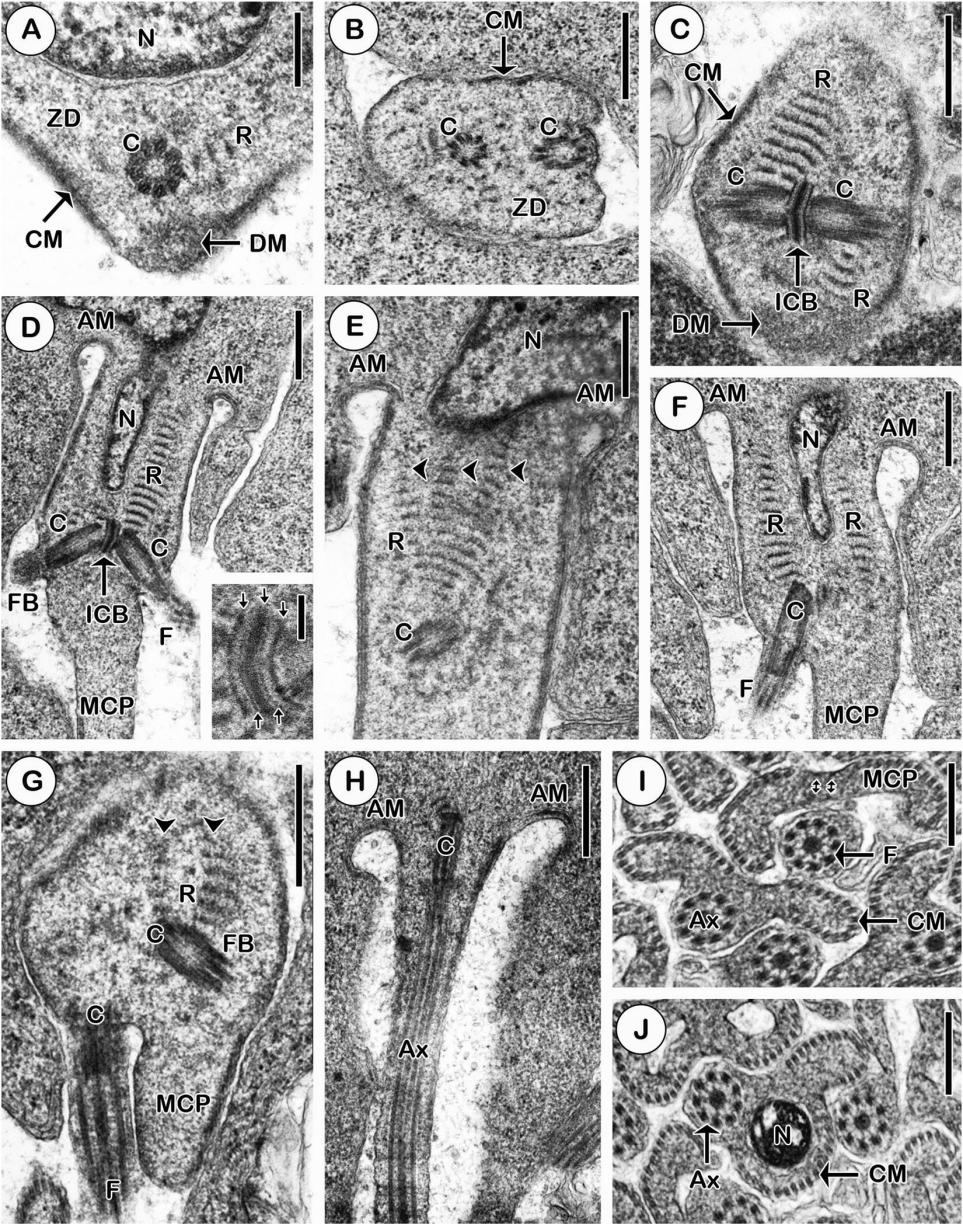
Early (**A**–**C**) and advanced stages (**D**–**J**) of spermiogenesis in *Khawia rossittensis*. (**A)** Early stage spermiogenesis with the zone of differentiation (ZD) containing dense material (DM), centriole (**C**) associated with striated root (R) situated tangential to the nucleus (**N**) and peripheral cortical microtubules (CM). **(B)** Cross section of the zone of differentiation with the two centrioles and cortical microtubules. (**C**) Typical striated roots of the two centrioles are situated opposite each other in the early stage of spermiogenesis. ICB - intercentriolar body. **(D)** Centrioles with the growing flagella (**F**) or flagellar buds (FB) rotate synchronously towards the median cytoplasmic process (MCP). AM - arching membranes. Inset: a detail illustrates five electron-dense plates (arrows) and four electron-lucent layers (asterisk) of the ICB. **(E)** The centriole is jointed with one striated root (R), which is branched into three thinner arms (arrowheads). **(F)** Penetration of the nucleus (N) into the MCP. **(G)** The flagellum (**F**) and the MCP are situated in a parallel way in the advanced stage of spermiogenesis. Note the unusual bifurcation (arrowheads) of the striated root (R). **(H)** The flagellum is fully incorporated into the MCP at the final stage of spermiogenesis. **(I)** Cross section showing the position of the free flagellum before and after proximodistal fusion. small arrows – attachment zones. **(J)** Cross section of young spermatid with the nucleus. Scale bars = 250 nm **(A)**; 500 nm **(B**–**H)**; 100 nm **(D** inset**)**; 330 nm **(I**,**J)**.

**Figure 2:**
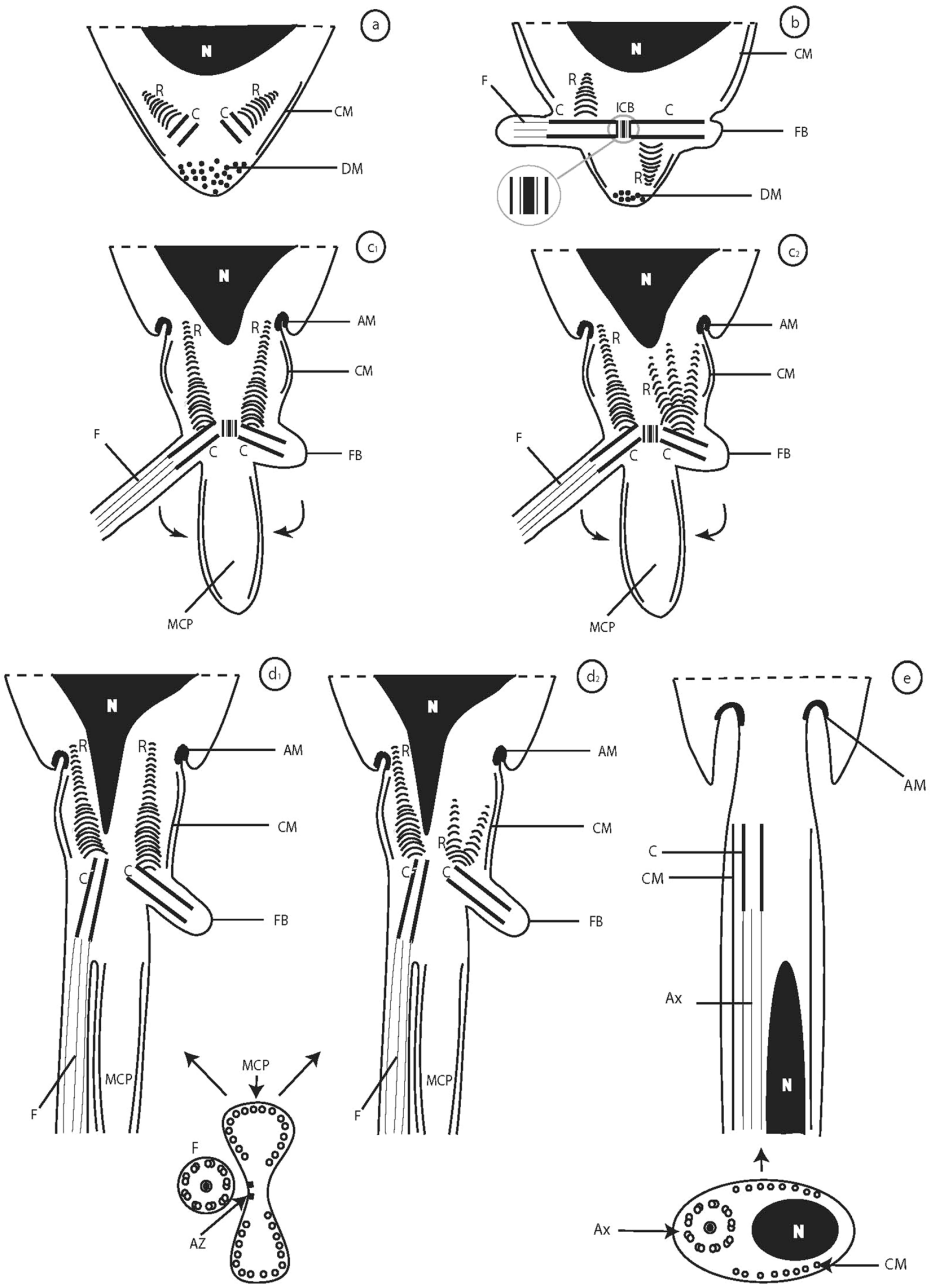
Schematic reconstruction of the early (**a**,**b**), advanced (**c**_**1**_–**d**_**2**_), and final (**e**) stages of spermiogenesis in *K*. *rossittensis*. AM - arching membranes, Ax - axoneme, AZ - attachment zones, C - centriole, CM - cortical microtubules, DM - dense material, F - flagellum, FB - flagellar bud, ICB - intercentriolar body, MCP - median cytoplasmic process, N - nucleus, R - striated root.

The very early stage of spermiogenesis in *Khawia rossittensis* is marked by the formation of the zone of differentiation (Fig. 1A,B). This area contains cortical microtubules, apical dense material and two centrioles associated with striated roots, which are oriented tangential to the apex of the nucleus (Figs 1A and 2a). Atypically, the striated roots are situated opposite of each other (Figs 1C and 2b). In advanced stages of spermiogenesis, a typical position of striated roots can be observed (Figs 1D,F and 2c_1_,d_1_). A remarkable feature is represented by the intercentriolar body, which comprises five electron-dense and four electron-lucent layers (Fig. 1D inset, 2b). The electron-dense plates are of different thicknesses: the thickest plate is the central plate, the less thick plates are the two peripheral plates, and the thinnest plates are the inner dense plates, which can be detected at higher magnification (Figs 1D inset, 2b). The branching of typical striated roots, where one typical root is branched into two or three arms, is another bizarre spermatology characteristic of *K*. *rossittensis* (Figs 1E,G and 2c_2_,d_2_). The formation of free flagella and flagellar buds is accompanied by their rotation towards the prolonging median cytoplasmic process (MCP) (Figs 1D,F,G and 2c_1_–d_2_). In the advanced stages of spermiogenesis, the two arching membranes are formed under the plasma membrane, and the nucleus migrates into the MCP (Figs 1D,E,F and 2c_1_–e). The free flagellum approaches (Figs 1G,I and 2d_1_,d_2_) and merges with the MCP during proximodistal fusion (Figs 1H–J and 2e). The attachment zones are present as two small islands of electron-dense material on the inner surface of the plasma membrane of the MCP (Figs 1I and 2d_1_,d_2_). At the end of spermiogenesis, the ring of arching membranes is constricted, and the spermatids are detached from the residual cytoplasm (Fig. 2e).

The mature spermatozoa of *K*. *rossittensis* are filiform cells tapered at both ends. These cells contain a single axoneme of trepaxonematan type 9 + ‘1’, cortical microtubules, cytoplasm with granules of glycogen and a nucleus. Five different regions with typical cytoarchitecture were recognized in ultrastructural studies of the male gametes of *K*. *rossittensis*.

**Figure 3:**
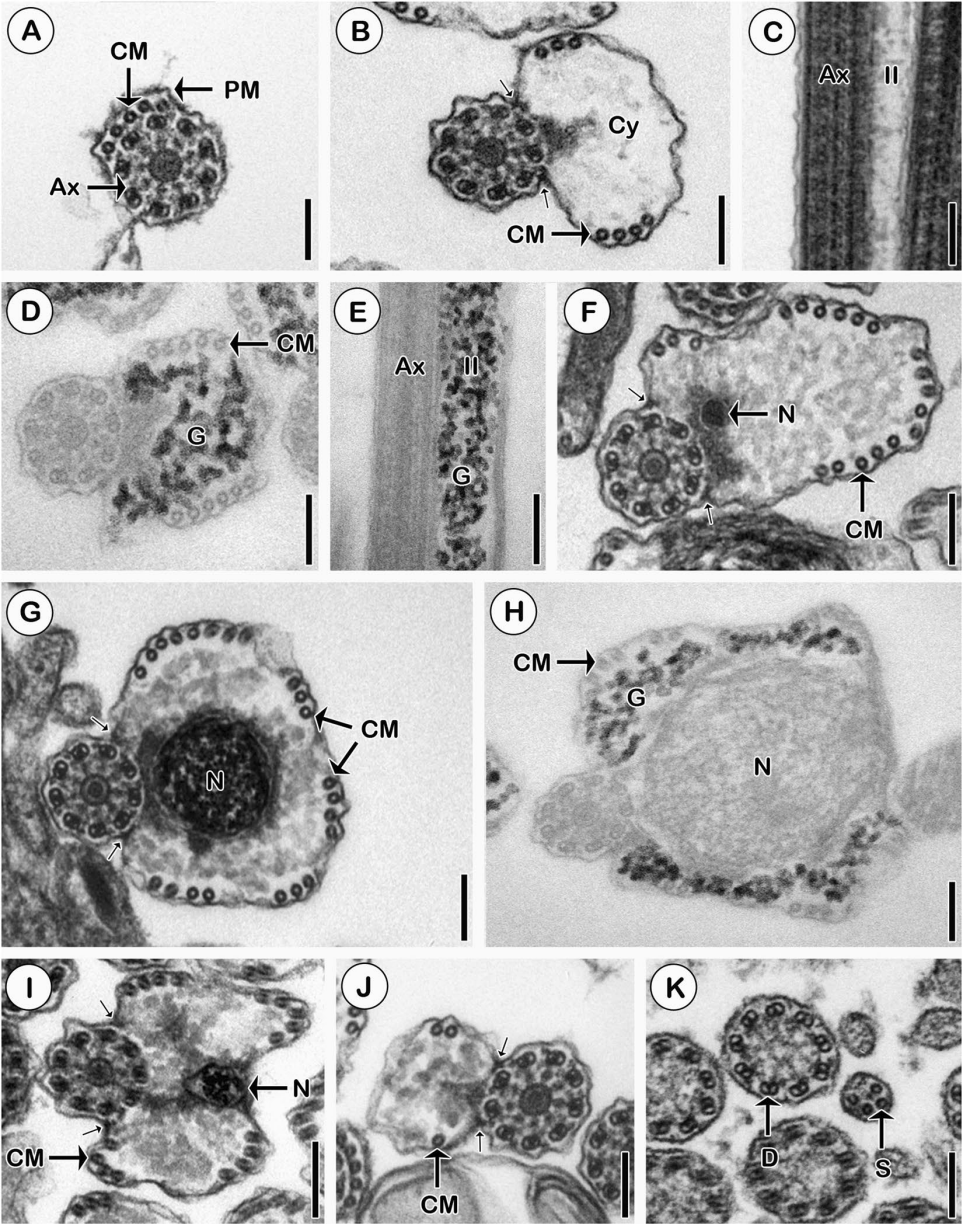
Fine structure of the mature spermatozoon of *K*. *rossittensis*. (**A)** Cross section of one axoneme (Ax) and five cortical microtubules (CM) beneath the plasma membrane (PM) in region I. **(B)** Cross section of region II with increasing volume of cytoplasm (Cy). Note one pair of attachment zones (small arrows). **(C)** Longitudinal section of region II. Note electron lucent cytoplasm, which contains glycogen granules (**G**) visualized using the Thiéry method (1967) **(D**,**E**). Cross sections showing various parts of the nucleated part of region III: anterior **(F)**, central **(G)**, central with glycogen **(H)** and posterior **(I)**. **(J)** Cross section of region IV displays strongly reduced cytoplasm and a few cortical microtubules (CM). **(K)** Cross section of region V of the mature spermatozoon indicates that the axoneme without the central electron dense complex unit is further disorganized into doublets (**D**) and singlets (**S**). Scale bars = 100 nm **(A**,**H)**; 125 nm **(B**,**F**,**G**,**J**,**K)**; 200 nm **(C**,**E**,**I)**; 80 nm **(D)**.

**Figure 4:**
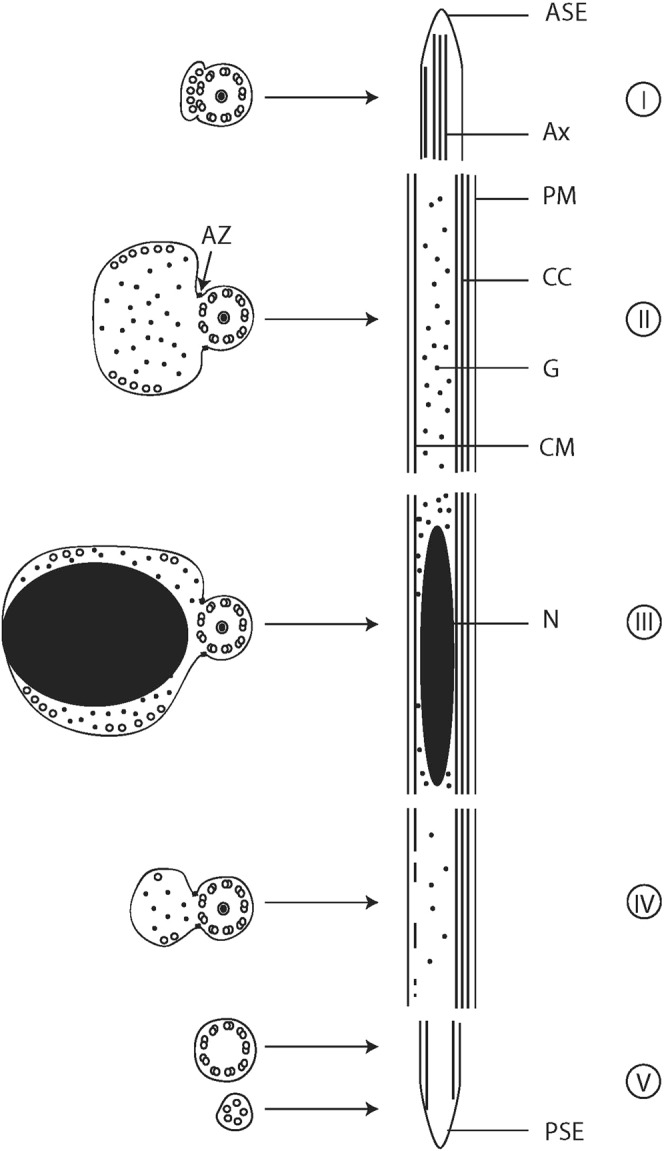
Schematic reconstruction of the mature spermatozoon of *K*. *rossittensis*. ASE - anterior spermatozoon extremity, Ax - axoneme, AZ - attachment zones, CC - central core, CM - cortical microtubules, G - glycogen, N - nucleus, PM - plasma membrane, PSE - posterior spermatozoon extremity; I, II, III, IV, V - five different regions of the male gamete.

Region I (Figs 3A and 4I), or the anterior part of the spermatozoon, contains one typical axoneme surrounded by a semi-arc of five cortical microtubules situated under the plasma membrane.

Region II (Figs 3B–E and 4II) exhibits an enlarged volume of cytoplasm and an increasing number of cortical microtubules (7–11). The cytoplasm is electron lucent in conventional ultrathin longitudinal and cross sections (Fig. 3B,C). The application of the Thiéry method (1967)^18^ enabled the detection of electron-dense granules of glycogen (Fig. 3D,E). One pair of attachment zones corresponds to the points of fusion of the free flagellum with the median cytoplasmic process during spermiogenesis (Figs 3B,F,G,I,J and 4II,III,IV).

Region III (Figs 3F–I and 4III) is a nucleated part of the spermatozoon. Anteriorly, the nucleus has a small diameter and is located near the axoneme (Fig. 3F). In the middle part of region III, the nucleus occupies the largest area of the cytoplasm, and the number of cortical microtubules increases up to 22 (Fig. 3G,H). The size of the nucleus and the number of cortical microtubules decrease posteriorly (Fig. 3I). Glycogen granules are situated in the peripheral cytoplasm of the spermatozoon (Fig. 3H).

Region IV (Figs 3J and 4IV) is characterized by a reduction in the cytoplasmic volume and the number of cortical microtubules (up to 3).

Region V (Figs 3K and 4V) or posterior part of the spermatozoon shows the axoneme without its central structure, i.e., nine peripheral doublets, which are continuously transforming into singlets.

**Figure 5:**
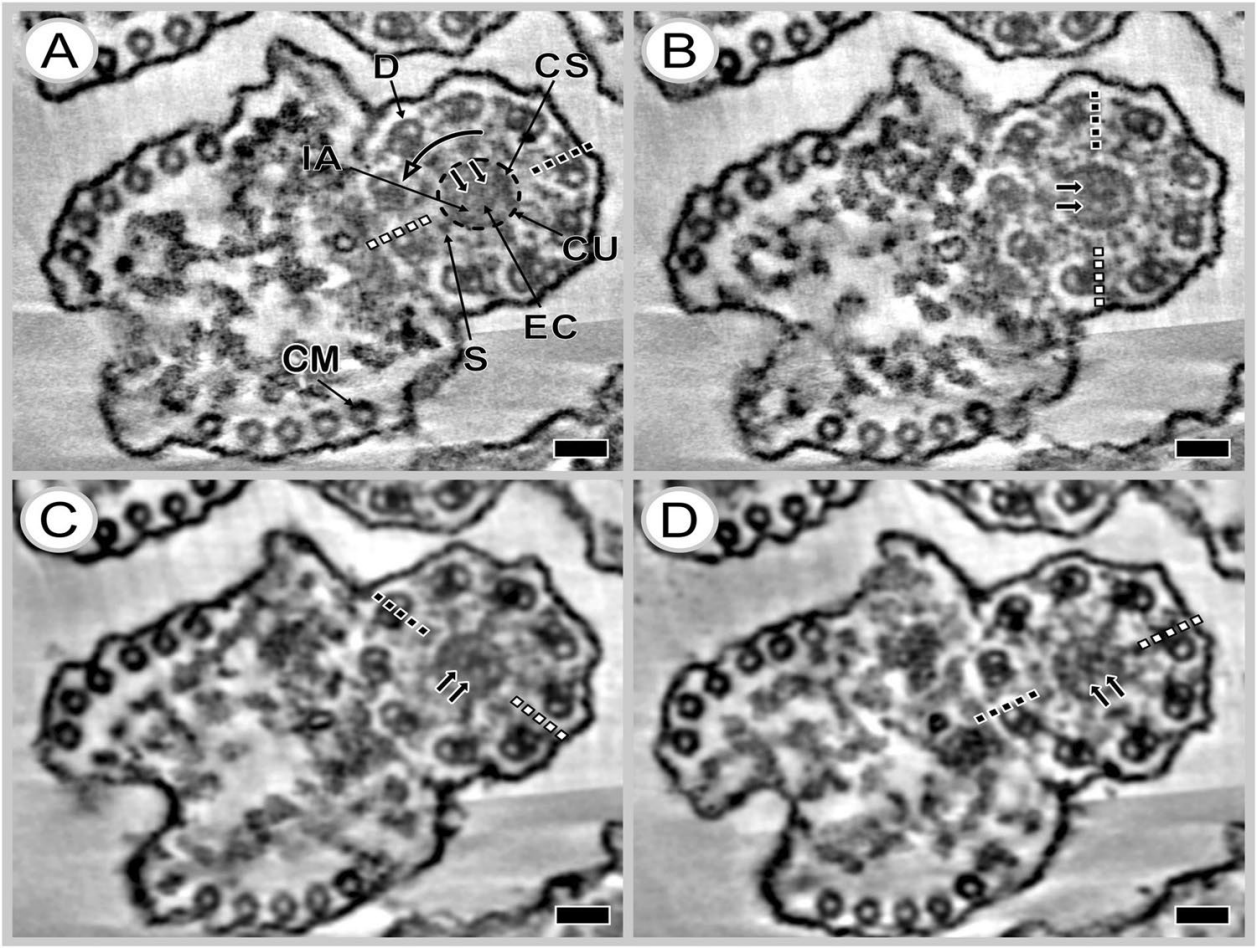
Electron tomography of the 9 + ‘1’ trepaxonematan axoneme of the mature spermatozoon of *K*. *rossittensis* illustrating the elliptical shape of the central electron-dense core with the two tubular structures (**A**–**D**). Tomogram virtual slices represent reconstructed volume ordered (**A**) first, (**B**) 13th, (**C**) 25th, (**D**) 37th from the bottom. **(A**,**B)** Tomograms show original slices. **(C**,**D)** Tomograms illustrate slices after application of a two-dimensional recursive Gauss filter using Amira software (Fisher Scientific). The lateral pixel size and thickness of each virtual slice are identical at 0.9 nm. Tilt series images were collected in the range ±70° with 0.8° increments. CM – cortical microtubules, CS - cortical sheath, CU - central unit, D – axonemal doublet microtubules, EC - central electron-dense core, IA - intermediate area, S – spike, small arrow - tubular structure. The dotted line indicates the orientation of EC, and the large curved arrow shows the direction of rotation of EC with increased placing in order of slices that represent the z-axis (height) in section volume. Scale bars = 50 nm.

**Figure 6:**
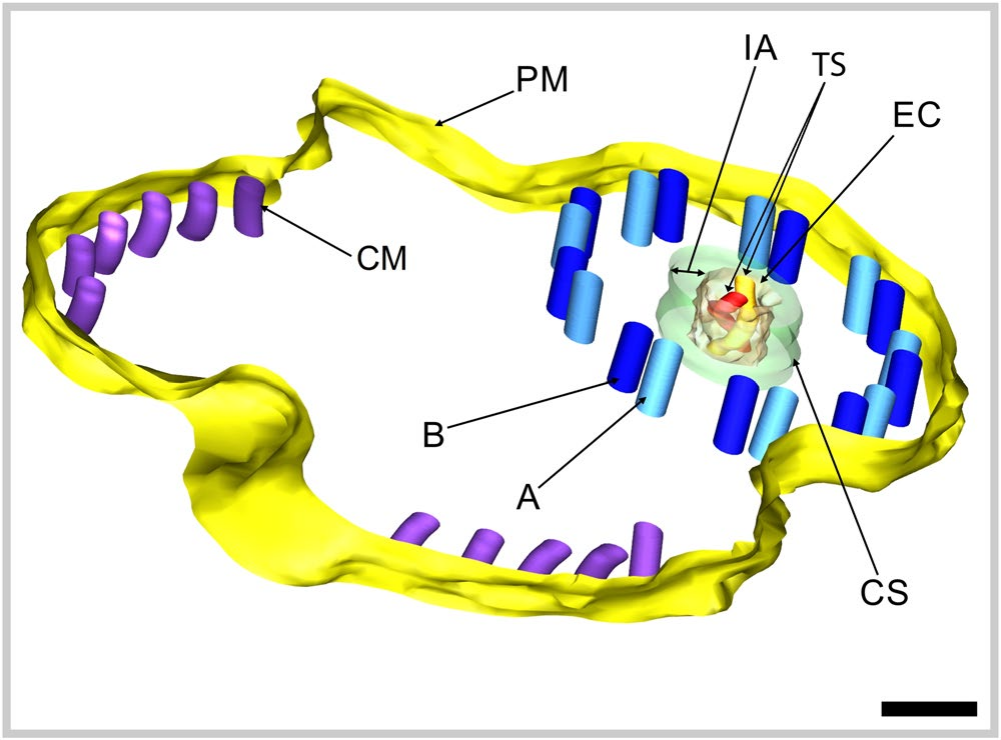
Three-dimensional model of the mature spermatozoon with 9 + ‘1’ trepaxonematan axoneme in *K*. *rossittensis*. The electron-dense core (EC) has a helical screw conveyor shape with two central tubular structures (TS). Tilt series images were collected in the range ±70° with 0.9° increments. A, B - A and B tubules of each axonemal doublet, CM - cortical microtubules, CS - cortical sheath, IA - intermediate area, PM - plasma membrane. Scale bar = 50 nm.

**Figure 7:**
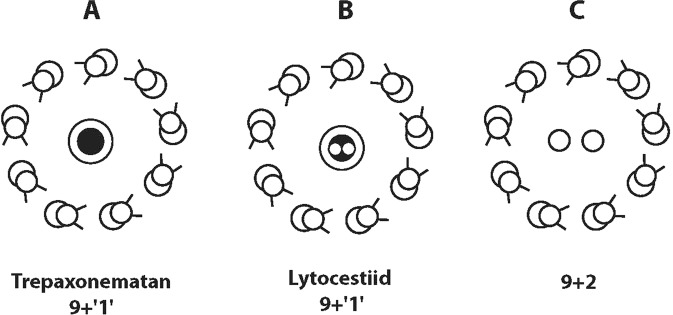
Diagram of transverse sections of the 9 + ‘1’ (**A**,**B**) and 9 + 2 (**C**) axonemal structures. Note a new trepaxonematan axonemal pattern represented by the two tubular structures in the central electron dense core of the mature spermatozoa in two lytocestid tapeworms studied to date **(B)**.

Electron tomography investigations of one-axoneme mature spermatozoon of *K*. *rossittensis* with the 9 + ‘1’ trepaxonematan structure were based on the analysis of a tomogram virtual slices with increasing Z-position (Fig. 5A–D). Our results showed that the central core unit (CU) is interconnected with nine peripheral axonemal doublets (D) by regularly spaced spokes (S). The central core unit consists of a central electron-dense core (EC) and electron-lucent intermediate area (IA), which is peripherally surrounded by an electron-dense cortical sheath (CS). The EC has a nearly elliptical shape and contains two tubular structures that are visible in all tomogram virtual slices as a white central electron-lucent area surrounded by a dark electron-dense border (white arrowhead in Fig. 5). The diameter of each tubular structure is 14.4 ± 1.4 nm (measured black-to-black border). The 3D model of central tubular structures was constructed by tracking the centre of the white area on each virtual section in the lateral position. This model illustrates the helical nature of the two tubular structures (Figs 6 and 7), and their rotation is counterclockwise (black arrow) with increasing Z-position of each virtual slice from the bottom (Fig. 5). The outer diameter of EC is 40.4 nm ± 1.6 nm and ~33.3 nm in the Z-axis direction and represents one-half period of screw (Fig. 5A–D).

## Discussion

Our observations on spermiogenesis in *Khawia rossittensis* revealed interesting ultrastructural features not described previously in traditional models of sperm formation in the Caryophyllidea^19,20^, e.g., apical electron-dense material and striated roots situated unusually in opposite direction in early stages, and rotation of both free flagella and flagellar buds. A branching of the striated root has not been described in the Caryophyllidea. A unique tubular doublet was detected using electron tomography in the central axonemal core of the spermatozoon with 9 + ‘1’ trepaxonematan structure in the present study.

Electron-dense material in the apical region of the zone of differentiation in the early stages of spermiogenesis has been observed in lytocestid tapeworm *Khawia armeniaca*^12^ for the first time within the order Caryophyllidea. Subsequently, this material was detected in other caryophyllidean species: *Wenyonia virilis*^21,22^, *Khawia sinensis*^13^, *Breviscolex orientalis*^23^, *Lytocestus indicus*^14^, *Hunterella nodulosa*^24^, *Caryophyllaeus laticeps*^25^, *Caryophyllaeides fennica*^11^, and *K*. *rossittensis* (present study). This character also appears in other cestode groups, e.g., Amphilinidea^26^, Spathebothriidea^27,28^, Diphyllobothriidea^29–32^, Bothriocephalidea^33–38^, and Proteocephalidea^39^.

The atypical arrangement of the two striated roots in opposite direction during the early stages of spermiogenesis as described in *K*. *rossittensis* in the present study has been reported in the lytocestid *Caryophyllaeides fennica*^11^, the caryophyllaeid *Caryophyllaeus laticeps*^25^, and the diphyllobothriidean *Diphyllobothrium latum*^29^. These spermatology findings may support the hypothesis based on molecular analysis that Caryophyllidea and Diphyllobothriidea are sister groups^40^. Another remarkable and unusual feature of Eucestoda is the branching of typical striated roots, where one typical root is branched into two or three arms. However, further observations are needed to elucidate the occurrence and/or importance of this unusual architecture of striated roots in cestodes.

The intercentriolar body (ICB), a phylogenetically important character, is considered to be a plesiomorphic feature of the Eucestoda^5,6^. Its composition may be closely related to the evolution of cestodes. The remarkable variability of this cell structure has been recorded in caryophyllidean cestodes of the family Lytocestidae. Here, the ICB is either absent, namely, in *Monobothrioides chalmersius*^10^ and *Atractolytocestus huronensis*^13^, or present and may consist of a variable number of electron dense layers, i.e., one in *K*. *armeniaca*^12^, three in *K*. *sinensis*^13^ and *L*. *indicus*^14^, or five in *K*. *rossittensis* (present study). Whereas the ICB of most caryophyllideans contains three electron-dense layers^21–25^, five electron-dense layers have been detected in *Archigetes sieboldi*^41^ and in *K*. *rossittensis* (present study). The latter pattern has also been reported in some diphyllobothriideans^29,30^ and trypanorhynchs^42^.

The rotation of both free flagella and flagellar buds to the median cytoplasmic process at 90° has not been described in traditional models of the spermiogenesis of the Caryophyllidea^10,19,20,22,43^. Subsequently, this feature was observed in some lytocestids^8,11–13^, caryophyllaeids^21,25,41^, capingentids^23^ and some evolutionarily higher non-cyclophillidean cestodes with one axoneme spermatozoa, i.e., tetraphyllideans^44^, tetrabothriideans^45^, or nippotaeniids^17^. The flagellar bud may represent a strongly reduced flagellum developed during the spermiogenesis of cestodes with two-axoneme spermatozoa, and consequently, the rotation of both free flagella and flagellar buds may indicate a derived stage of spermiogenesis in the Caryophyllidea^17^.

Glycogen is a branched polymer of glucose residues that presumably serves as an energy reserve for the motility of spermatozoa and other activities essential to the fertilization of ova^46^. An investigation of glycogen using a cytochemical method by Thiéry^18^ revealed the presence of glycogen granules in the cytoplasm of prenuclear, nuclear, and postnuclear regions in the mature spermatozoa of *K*. *rossittensis*. Similar results were reported in two other caryophyllideans, *C*. *laticeps*^25^ and *C*. *fennica*^11^. It should be noted, however, that intraaxonemal glycogen has not been detected in the mature spermatozoa of any caryophyllideans or Platyhelminthes with the 9 + ‘1’ trepaxonematan structure.

Our electron tomography analysis revealed unique two tubular structures in the centre of the electron-dense core of the 9 + ‘1’ trepaxonematan axoneme in the spermatozoon in *K*. *rossittensis*. A similar structure was described in the lytocestid *C*. *fennica* for the first time^15^. Virtual tomogram slices of both lytocestid cestodes showed the two central tubular structures, which may resemble two free parallel central singlet microtubules of the 9 + 2 axoneme of cilia and flagella, as described by Fawcett and Porter^47^ for the first time. On the other hand, the helical appearance of central doublet microtubules in cestodes (Fig. 6) does not support any hypothesis about the homology of these structures. Notably, the 9 + 2 axonemes are supposed to be almost ubiquitous structures, which are evolutionarily conserved and evolved in early eukaryotes nearly a billion years ago^48^. Our recent electron tomography findings have introduced a new model for the spermatozoon axoneme with a 9 + ‘1’ trepaxonematan structure, which, until recently, has been described exclusively in lytocestid caryophyllideans (Fig. 7).

The helicoidal nature of the central tubular doublet in *K*. *rossittensis* resembles the spiral shape of the central electron-dense core within the central cylinder of the single axoneme of the spermatozoon in nippotaeniid cestodes^17^. A helical substructure is supposed to be a more fundamental configuration than the derived protofibrilar substructure^49^.

The present study provides an original dataset of the novel spermatology features of lytocestid caryophyllidean tapeworms. The unique architecture of the 9 + ‘1’ axoneme of mature spermatozoa, as revealed by electron tomography, has been reported in only two lytocestids. Therefore, further studies of the spermatozoa of the tapeworms sensu stricto (Eucestoda) are urgently needed for the effective use of these new characters for the clarification of systematic and phylogenetic questions within the Platyhelminthes.

## Materials and Methods

### Parasite sampling

Live adult specimens of *Khawia rossittensis* (Szidat, 1937) were obtained from the intestine of prussian carp *Carassius gibelio* (Bloch, 1782) (Cypriniformes, Cyprinidae) captured from the Tisa River, Southeastern Slovakia. The fish were dissected using the standard methods described by Ergens and Lom^50^. All applicable institutional, national and international guidelines for the care and use of animals were followed and approved by the Animal Care and Use Committee of the Institute of Parasitology, Slovak Academy of Sciences, Košice (Slovakia). Tapeworms were cooled in a 0.9% NaCl solution and immediately processed for ultrastructural studies in the following way.

### Electron microscopy

The worms were cut into small pieces, fixed in 2.5% glutaraldehyde in 0.1 M sodium cacodylate buffer (pH 7.4) for 10 days at 4 °C, washed with 0.1 M sodium cacodylate buffer, postfixed in 1% OsO_4_ in 0.1 M cacodylate buffer for 2 hours at 4 °C, dehydrated in a graded alcohol series and embedded in Spurr epoxy resin. Semithin sections were cut with glass knives on an LKB Bromma 8880 ultramicrotome, stained with methylene blue and examined under a light microscope for the localization of the testes and vas deferens. Ultrathin sections were cut with a diamond knife on a Leica Ultracut UCT ultramicrotome, placed on copper grids and double stained with uranyl acetate and lead citrate. The grids were examined on a JEOL 1010 transmission electron microscope operating at 80 kV.

### Cytochemistry

Visualization of glycogen granules in male gametes was carried out using the Thiéry method^18^, the periodic acid-thiosemicarbazide-silver proteinate (PA-TSC-SP) technique, using the following protocol: ultrathin sections were placed on gold grids, treated in 1% PA (20–25 minutes), washed with distilled water, processed in 1% TSC (40 minutes), washed with 10% acetic acid and distilled water, treated in 1% SP (30 minutes), and finally washed with distilled water and dried. Gold grids were examined on a JEOL 1010 transmission electron microscope operating at 80 kV.

### Electron tomography

Ultrathin sections of 100 nm thickness were placed on 300 mesh copper grids (SPI), and staining was performed using alcoholic uranyl acetate and lead citrate both for 15 minutes. Au nanoparticles 10 nm conjugated with protein-A (BBI) as fiducial markers were placed on both sides and the surfaces were covered by carbon. We performed electron tomography as a dual axis in the range ±70° with a tilt step of 0.8° by means of a JEOL 2100F transmission electron microscope (TEM) equipped with a motorized tilt stage and a Orius SC 1000 Gatan camera automatically controlled by SerialEM software^51^. Tomogram reconstruction and model segmentation were performed with the IMOD software package^52^. The reconstructed tomogram with an isotropic voxel size of 0.9 nm was filtered for noise reduction by a 3D median filter and stored as an MRC file^53^. The contrast of virtual tomogram slices was enhanced by using an ImageJ plugin Enhanced Local Contrast (CLAHE) (imagej.net/Enhanced_Local_Contrast_(CLAHE)).

## Data Availability

The datasets generated and/or analysed during the current study are available from the corresponding authors on reasonable request.
